# Endocrine profiles and cycle characteristics of infertile 17α-hydroxylase/17,20-lyase Deficiency Patients undergoing assisted Reproduction Treatment: a retrospective cohort study

**DOI:** 10.1186/s13048-023-01190-6

**Published:** 2023-06-14

**Authors:** Ping Pan, Lingyan Zheng, Jia Huang, Xiaoli Chen, Renmin Ni, Qingxue Zhang, Dongzi Yang, Yu Li

**Affiliations:** 1 Reproductive Medicine Centre, Department of Obstetrics and Gynecology, Sun Yat-sen Memorial Hospital, Sun Yat-sen University, 107 West Yan Jiang Road, Guangzhou, 510120 Guangdong China; 2 Department of Reproductive Medicine, Kunming Angel Women’s and Children’s Hospital, Kunming, Yunnan China

**Keywords:** 17α-hydroxylase/17,20-lyase deficiency, Congenital adrenal hyperplasia, Infertility, Pregnancy, Assisted reproductive technique

## Abstract

**Background:**

17α-hydroxylase/17,20-lyase deficiency (17-OHD) is a rare form of congenital adrenal hyperplasia caused by *CYP17A1* gene variants. Female patients with 17-OHD demonstrate a broad clinical spectrum, including oligomenorrhea or amenorrhea and infertility, often as the sole manifestation. However, no spontaneous pregnancies in affected women have been reported.

**Objective:**

This retrospective cohort study aimed to explore the endocrine characteristics and assisted reproductive technique (ART) performance in women with 17-OHD.

**Methods:**

Five women were referred for primary infertility in a university-affiliated hospital over an eight-year period. The endocrine profiles and cycle characteristics during a total of nine cycles of ovarian stimulation and eight cycles of frozen-thawed embryo transfer (FET) were described in details.

**Results:**

Three cases had homozygous variants and two cases had compound heterozygous variants, including one novel missense variant (p.Leu433Ser) in the *CYP17A1* gene. Despite dual-suppression of progesterone (P) production by glucocorticoid and gonadotropin releasing hormone agonist, gradually increased P level, relatively low estradiol concentrations and thin endometrium were observed, negating fresh embryo transfer. During FET cycles, appropriate treatment resulted in low serum P levels and adequate endometrial thickness, leading to four live births.

**Conclusions:**

Our findings demonstrate that continuous elevation of serum P during follicular growth impairs endometrial receptivity, the likely cause of female infertility in 17-OHD. Therefore, female infertility caused by 17-OHD is suggested as an indication for freeze-all strategy, with promising reproductive prognoses following segmented ovarian stimulation and FET treatment.

## Introduction

Congenital adrenal hyperplasia (CAH) is a group of autosomal recessive disorders characterized by defects in enzymes or proteins involved in cortisol biosynthesis. 17α-hydroxylase/17,20-lyase deficiency (17-OHD) is a rare form of CAH arising from variants in *CYP17A1*, with an estimated incidence of 1 in 50,000 ~ 100,000 individuals [1, 2]. Worldwide, 17-OHD represents approximately 1% of CAH [3]. However, 17-OHD has been more frequently reported in the Chinese population, and thus considered to be the second most common form of CAH in China [4–7]. *CYP17A1* variants impair cytochrome P450 17α-hydroxylase (P450c17) enzyme expression in the adrenal gland and gonads, resulting in cortisol and sex steroid deficiency, in combination with mineralocorticoid excess [1]. The typical presentations of 17-OHD in 46, XX patients include primary amenorrhea, absent secondary sexual development, low-renin hypertension, and hypokalemia [7–9]. It has been reported that some females with 17-OHD have spontaneous menses and pubertal development, but suffer from oligomenorrhea, ovarian cysts and infertility [3, 9]. To the best of our knowledge, no spontaneous pregnancies have been described in affected females due to disordered steroidogenesis. To date, assisted reproductive techniques (ART) seem to be the preferred therapeutic approach to overcome reproductive barriers in such patients [7, 10–14].

Considering infertility impacts quality of life, and 17-OHD is a rare disorder, fertility potential is still poorly understood in this population. Therefore, we conducted a retrospective cohort study to explore the endocrine characteristics and ART performance in females with 17-OHD, in order to expand the management of female infertility.

## Materials and methods

In this retrospective cohort study, five unrelated female patients with 17-OHD undergoing ART treatment at the Reproductive Medicine Centre (Sun Yat-Sen Memorial Hospital, Sun Yat-Sen University) between January 2014 and December 2021 were identified. Written informed consent was obtained for ART from each patient. Moreover, this study was approved by the ethics committee of Sun Yat-sen Memorial Hospital, Sun Yat-sen University (SYSKY-2022-143-01).

### Clinical and laboratory evaluation

Relevant symptoms, family history and comprehensive fertility examinations of 5 patients were reviewed in detail. Karyotype analysis and anthropometric measurements including height, weight, and blood pressure were conducted. In addition to routine physical examination, the presences of axillary and pubic hair as well as breast and genitalia development were noted. Hormonal and biochemical profiles included follicle stimulating hormone (FSH), luteinizing hormone (LH), prolactin, estradiol (E_2_), progesterone (P), testosterone (T), anti-Müllerian hormone (AMH), 17-hydroxyprogesterone (17-OHP), dehydroepiandrosterone sulfate (DHEAS), androstenedione, thyroid stimulating hormone (TSH), adrenocorticotropic hormone (ACTH), serum cortisol, serum sodium, and serum potassium.

### *CYP17A1* gene analysis

Genomic DNA was extracted from peripheral blood sample using a commercial kit (Qiagen, Germany). The reference sequence of *CYP17A1* was retrieved from the UCSC Genome Browser on Human Feb. 2009 Assembly (hg19) (http://genome.ucsc.edu). All coding regions and flanking introns were amplified by genomic DNA polymerase chain reaction (PCR) using specific primers designed by Oligo 6.0 (http://www.oligo.net/downloads.html). The 2015 American College of Medical Genetics and Genomics (2015 ACMG) standards and guidelines were used to classify the variations [15]. Variant annotation and pathogenicity prediction were performed using PolyPhen-2 (http://genetics.bwh.harvard.edu/pph2/) and MutationTaster (http://www.mutationtaster.org/).The Genome Aggregation Database (gnomAD) (http://gnomad.broadinstitute.org/), dbSNP (https://www.ncbi.nlm.nih.gov/snp/) and 1000 Genomes (1000G) browser (https://www.internationalgenome.org/) were used to determine allele frequencies of variants.

### Fertility treatment

All patients underwent conventional in vitro fertilization (IVF) treatments depending on normal profiles of partners. Variable controlled ovarian stimulations (COS) were performed in our clinic based on features of ovarian reserve, such as gonadotropin releasing hormone agonist (GnRH-a) long protocol, GnRH antagonist protocol, luteal phase stimulation and progestin-primed ovarian stimulation (PPOS). However, two patients had attempted IVF treatments using ultra-long GnRH-a protocol and short-acting GnRH-a long protocol in other hospitals before referral to our clinic. All available embryos were cryopreserved by vitrification. In frozen-thawed embryo transfer (FET) cycles, patients were given oral glucocorticoids and GnRH-a injections to suppress progesterone excess from both adrenal and gonadal sources. When progesterone value was well controlled in the range of follicular phase, artificial endometrial preparation was started with oral estradiol valerate at a dosage of 4-6 mg/d (Progynova; Bayer Schering Pharma, France). When endometrial thickness exceeded 7 mm, endometrial transformation was applied with progesterone administration until pregnancy testing. A maximum of three frozen-thawed embryos were transferred. At the time of transfer, the use of oral glucocorticoids was stopped. If pregnancy was achieved, luteal support was maintained until 12 weeks gestation.

## Results

### Clinical manifestations

As shown in Table 1, barring Patient 4 (P4), all patients had spontaneous menarche, three whom complained of prolonged menstrual bleeding. Because of primary amenorrhea, P4 received glucocorticoid therapy to establish menstruation after the diagnosis of 17-OHD, but her menses onset was characterized by oligomenorrhea. All cases were referred for primary infertility, and comprehensive infertility evaluations revealed little except bilateral tubal obstruction in Patient 3 (P3). Three patients (P1, P2, P5) presented with recurrent ovarian cysts. All patients developed normal secondary sexual characteristics, however, pubic and axillary hair were scarce or absent. Unlike individuals with classic 17-OHD, they exhibited normal blood pressure.

**Table 1:** Clinical manifestations of the study patients

	Patient 1	Patient 2	Patient 3	Patient 4	Patient 5
Referral time	February 2014	March 2017	May 2017	January 2019	May 2019
Age, y	29	28	31	29	28
Menarche	Spontaneous, 14 years	Spontaneous, 14 years	Spontaneous, 14 years	Primary amenorrhea	Spontaneous, 14 years
Menses pattern	Regular cycle with menostaxis	Irregular cycle with menostaxis	Regular cycle	Irregular cycle	Irregular cycle with menostaxis
Gynecological symptoms	Recurrent ovarian cysts	Recurrent ovarian cysts	None	None	Repeated endometrial cavity fluid and ovarian cysts
Family history	A fertile brother, a twin sister with 17-OHD	No special	A fertile brother	A fertile sister, an unmarried brother	A sister with irregular menses
Infertility type	Primary	Primary	Primary	Primary	Primary
Duration of infertility, y	7	4	7	3	6
Tubal patency assessment	Bilateral Patency	Bilateral Patency	Bilateral obstruction	Bilateral Patency	Bilateral Patency
Hysteroscopy	Normal uterine cavity	Not examined	Normal uterine cavity	Not examined	Intrauterine adhesion
Partner’s semen analysis	Normal	Normal	Normal	Normal	Normal
Treatment experience before referral	Three cycles of IUI	Ovulation detection	Two IVF cycles	One IVF cycle	Misdiagnosis of PCOS, repeated hysteroscopy
Height, cm	158	163	162	168	168
Weight, kg	63	56	64	54	64
BMI (kg/m^2^)	25.24	21.08	24.39	19.13	22.68
Blood pressure (mmHg)	121/76	110/77	120/77	100/73	106/67
Axillary hair	Sparse	No	No	No	No
Tanner stage	B5PH2	B5PH1	B5PH2	B5PH1	B5PH1
Genitalia	Normal	Normal	Normal	Normal	Normal
Karyotype	46, XX	46, XX	46, XX	46, XX	46, XX

*17-OHD, 17α-hydroxylase/17,20-lyase deficiency; B, breast; BMI, body mass index; IUI, intrauterine insemination; IVF, in vitro fertilization, PCOS, polycystic ovarian syndrome; PH, pubic hair*

### Hormonal profiles

Patient 1 (P1) was diagnosed with 17-OHD after ovarian stimulation in our clinic, whereas the remaining patients had earlier diagnoses and had a history of glucocorticoid treatment prior to referral. As shown in Table 2, several hormone features were common amongst all patients. Pituitary hormone profiles including serum FSH, LH, prolactin and TSH were normal in most cases, while mild LH elevation was observed in P2 and P5. Serum E_2_, T, DHEA and androstenedione levels were in the low-normal range. Follicular phase progesterone was high in all patients except P4, who was on glucocorticoid replacement therapy. Basal 17-OHP levels were within the reference range of follicular phase. In accordance with the clinical features of each patient, ACTH, cortisol and electrolytes were within normal ranges.

**Table 2:** Hormonal profiles of the study patients

	Patient 1	Patient 2	Patient 3	Patient 4	Patient 5	Normal range
Basal FSH (IU/L)	7.58	9.9	5.84	3.38	8.28	3.85–8.78
Basal LH (IU/L)	3.38	15.05	2.82	3.87	13.67	2.12–10.89
Prolactin (ug/L)	28.37	16.74	20.14	8.43	10.28	3.34–26.72
Basal E_2_ (ng/L)	67	< 20	39	27	28	27–122
Testosterone (nmol/L)	0.50	< 0.35	< 0.35	< 0.35	< 0.35	0.35–2.6
Basal Progesterone (ng/ml)	13.19	3.13	5.23	0.93*	15.88	0.31–1.52
17-OHP (ng/ml)	1.04	0.22	1.65	NR	1.11	0.10–1.40
DHEA-S (ug/dL)	710.03	67.80	86.42	NR	19.85	400–2170 (P1 P2,P3,), 98.8–340 (P5)
Androstenedione (ng/ml)	0.72	NR	NR	0.17	0.29	1.86–5.44 (P1), 0.75–3.89 (P4,P5)
TSH (mIU/L)	1.831	0.468	1.436	2.270	1.575	0.550–4.780
ACTH 8am, pg/ml	34	NR	28.8	NR	NR	0–46
ACTH 4pm, pg/ml	27	NR	NR	NR	NR	
Cortisol 8am, nmol/L	206.30	NR	351.00	NR	NR	118.6–618
Cortisol 4pm, nmol/L	109.48	NR	NR	NR	NR	
Serum potassium (mmol/L)	3.84	NR	4.27	4.54	NR	3.5–5.3
Serum sodium (mmol/L)	142.5	NR	144	141.9	NR	137–147

*17-OHP, 17α-hydroxyprogesterone; ACTH, adrenocorticotropic hormone; AMH, anti-Müllerian hormone, DHEA-S, dehydroepiandrosterone sulfate; E*_*2*_, estradiol; FSH, follicle stimulating hormone; LH, luteinizing hormone; NR, not recorded; SHBG, sex hormone-binding globulin; TSH, thyroid stimulating hormone.^*^Patient 4 took glucocorticoid

### Variant detection in *CYP17A1*

**Fig. 1:**
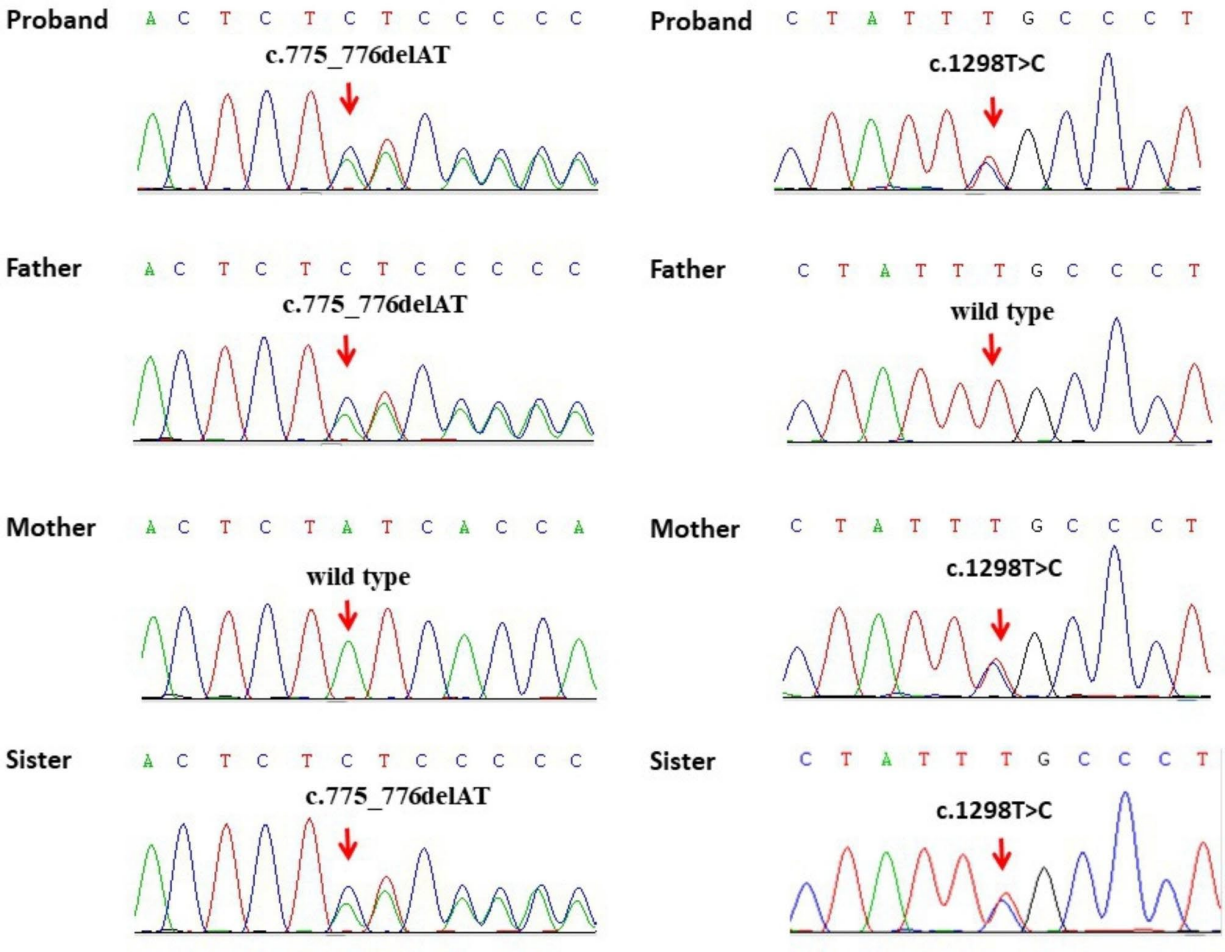
The sequencing chromatogram of Patient 1

Sanger sequencing revealed six variants in *CYP17A1* (Table 3). Three individuals had homozygous variants and two were compound heterozygotes. With the exception of P1 (Fig. 1), the remaining patients’ genetic reports came from other hospitals. In P1, the novel variant c.1298T > C was unreported in dbSNP, and 1000 genomes database. The minor allele frequency (MAF) in gnomAD was very low (0.000007). The c.1298T > C missense variant, predicted to result in loss of both 17α-hydroxylase and 17,20-lyase was designated damaging by *in silico* predictive tools, PolyPhen-2 and Mutation Taster. In P3, the variant c.985_987delinsAA results in a frameshift, potentially impacting splicing. The variant c.1346G > A was reported in dbSNP (rs752164207) and gnomAD at a low frequency (0.00000-0.000008). *In silico* analysis of the 1346G > A variant by PolyPhen-2 and Mutation Taster, deemed it to be probably damaging and disease causing. The same position variation c.1346 was included in HGMD and showed pathogenic (HGMD Accession Number: HM0669).

**Table 3:** The variants in *CYP17A1* of the study patients

	Parental analysis	variants	variant type	HGMD ID	PolyPhen-2	MutationTaster
Patient 1	PA	c.775_776delAT (p.Ile259Hisfs*15)	deletion	HD030003	NA	Disease causing
	MA	c.1298T > C(p.Leu433Ser)	missense	NA	Probably damaging	Disease causing
Patient 2	PA	c.1226 C > G(p.Pro409Arg)	missense	CM012743	Probably damaging	Disease causing
	MA	c.1226 C > G(p.Pro409Arg)	missense	CM012743	Probably damaging	Disease causing
Patient 3	PA	c.985_987delinsAA(p.Tyr329Lysfs*90)	indel	HX030003	NA	Disease causing
	MA	c.1346G > A(p.Arg449His)	missense	NA	Probably damaging	Disease causing
Patient 4	PA	c.1486 C > T(p.Arg496Cys)	missense	CM920226	Probably damaging	Disease causing
	MA	c.1486 C > T(p.Arg496Cys)	missense	CM920226	Probably damaging	Disease causing
Patient 5	PA	c.1226 C > G(p.Pro409Arg)	missense	CM012743	Probably damaging	Disease causing
	MA	c.1226 C > G(p.Pro409Arg)	missense	CM012743	Probably damaging	Disease causing

*PA, paternal allele; MA, maternal allele; NA, not data. HGMD*:https://www.hgmd.cf.ac.uk/ac/index.php

### Cycle characteristics of ovarian stimulation

Over a span of eight years, 5 patients underwent 9 cycles of COS in total (Table 4). Besides poor ovarian response in P4, the majority of patients showed normal ovarian response using GnRH-a or GnRH antagonist protocol. With exogenous gonadotropin stimulation, multiple follicles matured, but an abnormal sex hormone pattern was noted throughout the cycle, characterized by gradually increased follicular phase P level and relatively low E_2_ concentration. Late follicular-phase E_2_ was disproportionate compared to the number of dominant follicles. Overall, thin endometria were observed during ovarian stimulation. P3 had adequate endometrial thickness as E_2_ level on human chorionic gonadotropin (HCG) trigger day reached over 500ng/L. In all patients, ovarian stimulation resulted in available oocytes (ranging from 1 to 16) and viable embryos (ranging from 0 to 7). High P levels and inappropriate endometrial thickness negated fresh embryo transfer and all available embryos were cryopreserved.

**Table 4:** Controlled ovarian stimulation cycles of the study patients

	Patient 1	Patient 2	Patient 3		Patient 4				Patient 5
AFC	9	15	15		14				32
AMH (ng/ml)	3.19	9.06	2.52		2.69				13.35
Ovarian stimulation	May 2014	August 2017	June 2017	November 2017	February 2019	April 2019	April 2019	July 2019	September 2019
COS protocol	Luteal-phase GnRH-a long protocol	Follicular-phase GnRH-a long protocol	GnRH antagonist	Luteal-phase GnRH-a long protocol	PPOS	PPOS	Luteal phase stimulation	PPOS	Follicular-phase GnRH-a long protocol
LH suppression	Triptorelin 1.25 mg	Leuprorelin 3.75 mg	Ganirelix 0.25 mg/d	Triptorelin 1.87 mg	MPA 12 mg/d, Cetrotide 0.25 mg/d	MPA 12 mg/d	None	Femostone 4 mg/d, MPA 12 mg/d	Leuprorelin 3.75 mg
Glucocorticoid	None	DXM 0.75 mg/d	DXM 0.375 mg/d	DXM 0.375 mg/d	DXM 0.75 mg/d	DXM 0.75 mg/d	DXM 0.75 mg/d	DXM 0.75 mg/d	DXM 0.75 mg/d
E_2_ on initiation day, ng/L	< 20	< 20	26	< 20	< 20	28	< 20	< 20	< 20
P on initiation day, ng/ml	8.44	6.17	0.41	1.19	0.46	3.02	4.71	0.45	0.12
Duration of stimulation, days	15	11	11	12	9	None	9	10	9
Total gonadotropin dose, IU	4125	1650	3100	3600	1050	None	1500	3300	1200
Follicles ≥ 14 mm on HCG day	12	7	10	18	3	1	5	6	13
E_2_ on HCG day, ng/L	296	57	636	518	43	< 20	46	55	106
P on HCG day, ng/ml	17.37	6.23	3.43	8.31	5.56	2.31	15.36	5.92	6.25
Endometrial thickness on HCG day, mm	4.8	6.0	9.1	8.0	6.2	6.6	5.7	7.3	2.6
Number of retrieved oocytes	12	9	11	16	3	1	5	8	15
Number of available embryos	5	5	2	2	1	0	2	2	7

*AFC, antral follicle count; AMH, anti-Müllerian hormone; COS, controlled ovarian stimulation; DXM, dexamethasone; E*_*2*_, *estradiol; GnRH-a, gonadotropin releasing hormone agonist; HCG, human chorionic gonadotropin; LH, luteinizing hormone; MPA, medroxyprogesterone acetate; P, progesterone; PPOS, progestin-primed ovarian stimulation*

### Cycle characteristics and pregnancy outcomes of frozen embryo transfer

In total, 8 FET cycles were conducted (Table 5). With dual-suppression by oral glucocorticoids and GnRH-a administration, serum P levels on transformation day were usually very low or undetectable. For P5, dexamethasone was administrated only during FET cycle, since there was no dominant follicle growth and her P concentration was kept at a stable-low level. Adequate endometrial thickness was found after exogenous oestrogen supplementation, ranging from 7.8 to 17.0 mm. Two embryos were transferred in six FET cycles, four FET cycles developed into live births. FET failed in P2 mainly due to poor control of serum P level in FET cycle. Despite having three additional frozen embryos in our centre, she has not returned for further embryo transfer.

**Table 5:** Frozen-thawed embryo transfer and pregnancy outcomes of the study patients

	Patient 1		Patient 2	Patient 3	Patient 4			Patient 5
GnRH-a administration	Triptorelin 3.75 mg	Triptorelin 3.75 mg, twice	Triptorelin 3.75 mg	Triptorelin 3.75 mg	Triptorelin 1.87 mg	Triptorelin 3.75 mg	Leuprorelin 3.75	None
Glucocorticoid	DXM 0.375 mg/d	DXM 0.375 mg/d	DXM 0.75 mg/d	DXM 0.75 mg/d	DXM 0.75 mg/d	DXM 0.75 mg/d	DXM 0.75 mg/d	DXM 0.75 mg/d
Duration of estradiol valerate administration, days	11	9	10	15	12	12	11	14
Endometrial thickness on transformation day, mm	7.8	8.2	11.2	9.0	12.1	12.0	17.0	11.9
E_2_ on transformation day, ng/L	None	None	221	220	114	309	177	261
P on transformation day, ng/ml	None	0.18	3.10	0.21	< 0.1	< 0.1	< 0.1	0.22
Number of embryo transferred	2	2	2	3	2	1	2	2
Pregnancy outcome	Singleton pre-term live birth, vaginal delivery.	No pregnancy	No pregnancy	Singleton pre-term live birth, caesarean section.	No pregnancy	Biochemical pregnancy	Singleton full-term live birth, caesarean section.	Singleton full-term live birth, caesarean section.

*DXM, dexamethasone; E*_*2*_, *estradiol; GnRH-a, gonadotropin releasing hormone agonist; P, progesterone*

## Discussion

This study describes our centre’s experience in managing 5 cases of infertility in women with 17-OHD (46, XX). Our study focused on ART performance in 17-OHD patients including fresh and frozen embryo transfer. Results demonstrated that the fertility potential of females with 17-OHD was promising, and segmented ovarian stimulation and FET significantly improved chances of successful live births.

Although 17-OHD was first described in 1966 [16] and despite global cases of 17-OHD, little is known about fertility in these patients. *CYP17A1* encodes the key enzyme P450c17, which mediates both 17α-hydroxylase and 17,20-lyase activities. Combined enzyme deficiency is the most common form of 17-OHD [2, 9]. To date, there is no specific definition for complete or partial 17-OHD, and the clinical manifestation is mainly based on residual enzyme activity. Sometimes the diagnosis is quite challenging as phenotype variability occurs. The five patients described herein were defined as partial 17-OHD, given that they presented with androgen and oestrogen deficiency without mineralocorticoid excess and developed normal female secondary sex characteristics without hypertension or hydro-electrolyte imbalance.

Besides the aforementioned hormonal abnormalities, ovarian cyst and cyst rupture provided clinical clues as to the diagnosis of 17-OHD in some female patients [14, 17–20]. Ovarian cyst formation was ascribed to high P level or elevated gonadotropins without an oestrogen-triggered LH surge [9, 21]. The gynecological complaints of three patients (P1, P2, P5) were consistent with previous studies, and fortunately, none required ovarian surgery. Furthermore, P5 had a misdiagnosis of polycystic ovarian syndrome (PCOS) and underwent repeated hysteroscopy due to refractory endometrial cavity fluid. She had primary infertility and no history of endometrial tuberculosis, or other underlying causes of endometrial cavity fluid. 17-OHD should be distinguished from PCOS, especially in cases with PCO-like ovaries, elevated LH, and good ovarian reserve.

*CYP17A1* is located on chromosome 10q24.32 and consists of eight exons, encoding a 508 amino acid protein. Until now, variants in *CYP17A1* (OMIM * 609,300) exceeding more than 150, have included point variants, indel variants, splice site alterations, and large deletions (9). In our study, genetic analyses revealed one novel missense variant c.1298T > C (p.Leu433Ser), which was recently reported in Chinese hypospadias patients with karyotype 46, XY [22]. All variants in this study were predicted deleterious or likely to be pathogenic. Therefore, establishing a genetic diagnosis aided clinical management and therapy.

Spontaneous pregnancy has not been documented in women with 17-OHD and primary infertility. In previous studies, ovarian histology confirmed numerous primary and secondary ovarian follicles [23, 24], but follicular maturation was arrested in some affected women [25]. Mild forms of 17-OHD such as seen in P1 and P3, were associated with regular menstruation and normal genitalia, but primary infertility still supervened. The impairment of reproductive capacity in 17-OHD is presumed to be multifactorial including impaired folliculogenesis, anovulation, thin endometria and cervical dysmucorrhea.

What’s more, in our study even with dual-suppression of P production by GnRH-a administration and ongoing glucocorticoid therapy during ovarian stimulation, serum P level increased during the follicular phase. The distinctive response to ovarian stimulation in 17-OHD proved to be quite a useful tool. Firstly, as follicles grow, endogenous progesterone biosynthesis is activated resulting in P overproduction, hence natural conception is scarce and failed ovulation induction without IVF has only ever been reported in the literature [26, 27]. Secondly, since fresh embryo transfer was out of the question in 17-OHD patients, the choice of ovarian stimulation regimen was tailored to account for ovarian reserve and menstrual cycle regularity. Nearly half the COS cycles in this study were GnRH-a long protocol, thus ART procedures were prolonged. In this respect, we would like to propose 17-OHD as an indication for a freeze-all strategy in IVF and suggest flexible COS options in this population, in order to shorten the time to pregnancy. Thirdly, the traditional timing of triggering final oocyte maturation is generally related to follicle size, growing follicle cohort, hormonal data, experience of previous cycles, etc. [28]. In 17-OHD, the criteria for triggering was mainly based on the size of leading follicles, not taking into account E_2_ or P levels.

We searched the literature and found that the reproductive prognosis of 17-OHD was acceptable, and ART overrode the steroid biosynthetic defect state and helped affected females conceive with their own gametes [7, 10–14]. The successful therapeutic achievements in our study were a direct result of segmented ovarian stimulation and FET. It was reported that hypoplastic uteri was found in some cases [20, 29, 30], and uterine dysfunction might be deemed to impair fertility. As for our results, all 5 cases showed adequate endometrial thickness in FET cycles after exogenous oestrogen supplement. In our experiences, fertility potential in most cases of partial 17-OHD will not be compromised if oestrogen treatment improves uterine volume and endometrial thickness.

In summary, our findings demonstrate that continuous elevation of serum P during follicle growth impairs endometrial receptivity, the main cause of female infertility in partial 17-OHD. We suggest that primary infertility caused by 17-OHD should be an indication for a freeze-all strategy, aided by segmented ovarian stimulation and FET treatment.

## Data Availability

The data in the current study can be found in the electronic medical record system of Reproductive Medicine Centre, Sun Yat-Sen Memorial Hospital, Sun Yat-Sen University.
